# ATF4 expression in thermogenic adipocytes is required for cold-induced thermogenesis in mice via FGF21-independent mechanisms

**DOI:** 10.1038/s41598-024-52004-8

**Published:** 2024-01-18

**Authors:** Sarah H. Bjorkman, Alex Marti, Jayashree Jena, Luis Miguel García-Peña, Eric T. Weatherford, Kevin Kato, Jivan Koneru, Jason Chen, Ayushi Sood, Matthew J. Potthoff, Christopher M. Adams, E. Dale Abel, Renata O. Pereira

**Affiliations:** 1 Fraternal Order of Eagles Diabetes Research Center and Division of Endocrinology and Metabolism, Roy J. and Lucille A. Carver College of Medicine, University of Iowa, 169 Newton Road, 4338 PBDB, Iowa City, IA 52242 USA; 2 Department of Obstetrics and Gynecology, Reproductive Endocrinology and Infertility, University of Iowa Hospital and Clinics, Iowa City, IA USA; 3 Department of Neuroscience and Pharmacology, Roy J. and Lucille A. Carver College of Medicine, University of Iowa, Iowa City, IA USA; 4 Division of Endocrinology, Metabolism and Nutrition, Department of Medicine, Mayo Clinic, Rochester, MN USA; 5 Department of Medicine, David Geffen School of Medicine, University of California, Los Angeles, Los Angeles, CA USA

**Keywords:** Physiology, Metabolism, Fat metabolism

## Abstract

In brown adipose tissue (BAT), short-term cold exposure induces the activating transcription factor 4 (ATF4), and its downstream target fibroblast growth factor 21 (FGF21). Induction of ATF4 in BAT in response to mitochondrial stress is required for thermoregulation, partially by increasing FGF21 expression. In the present study, we tested the hypothesis that *Atf4* and *Fgf21* induction in BAT are both required for BAT thermogenesis under physiological stress by generating mice selectively lacking either *Atf4 (*ATF4 BKO*)* or *Fgf21* (FGF21 BKO) in UCP1-expressing adipocytes. After 3 days of cold exposure, core body temperature was significantly reduced in *ad-libitum*-fed ATF4 BKO mice, which correlated with *Fgf21* downregulation in brown and beige adipocytes, and impaired browning of white adipose tissue. Conversely, despite having reduced browning, FGF21 BKO mice had preserved core body temperature after cold exposure. Mechanistically, ATF4, but not FGF21, regulates amino acid import and metabolism in response to cold, likely contributing to BAT thermogenic capacity under ad libitum-fed conditions. Importantly, under fasting conditions, both ATF4 and FGF21 were required for thermogenesis in cold-exposed mice. Thus, ATF4 regulates BAT thermogenesis under fed conditions likely in a FGF21-independent manner, in part via increased amino acid uptake and metabolism.

## Introduction

Since the discovery of active brown adipose tissue (BAT) in adult humans, multiple studies have explored BAT thermogenic activation as a potential strategy for increasing energy expenditure and nutrient disposal to mitigate obesity and its complications^1–3^. More recently, BAT has been recognized as a secretory organ, promoting the release of batokines that may act in an endocrine manner to regulate systemic metabolism^4^. Indeed, several studies using β3-adrenergic agonists to activate BAT demonstrated changes in adiposity and improvements in glucose homeostasis^3,5,6^, however, this class of medication poses cardiovascular concerns^1^. Therefore, identifying new pathways that can be targeted to induce BAT’s thermogenic activity and secretory function, may lead to the discovery of new targets to improve metabolic health.

A recent study demonstrated that the activating transcription factor 4 (ATF4), the main effector of the integrated stress response (ISR), is induced in brown adipocytes in response to cold exposure following fasting, which correlates with a significant increase in fibroblast growth factor 21 (FGF21) circulating levels^7^. Furthermore, ATF4 overexpression in BAT is sufficient to improve thermogenesis in mice^8^. Similarly, our recent work showed that selective deletion of the mitochondrial fusion protein optic atrophy 1 (OPA1) in BAT induces the expression and secretion of fibroblast growth factor 21 (FGF21) as a batokine, via an ATF4-dependent mechanism. Activation of this ATF4-FGF21 axis was required to mediate an adaptive response characterized by elevated resting metabolic rates, and improved thermoregulation in OPA1-deficient mice^9^. Together, these studies suggest that ATF4 is likely to be operative in BAT under physiological circumstances and may regulate the secretion of FGF21 as a batokine. However, studies investigating ATF4 in BAT are few and were performed in whole-body knock out models and cultured adipocytes^10–14^, limiting our understanding of the specific roles of ATF4 in BAT. Moreover, although FGF21 is induced in BAT in response to thermogenic activation, whether ATF4 is required for this induction, and whether BAT-derived FGF21 is necessary for adaptive thermogenesis was yet to be investigated.

In the present study, we investigated the requirement of ATF4 and FGF21 induction in thermogenic adipocytes for adaptive thermogenesis by generating mice with selective deletion of either *Atf4* or *Fgf21* in UCP1-expressing adipocytes. Our data demonstrated that neither ATF4 nor FGF21 are required for the activation of the thermogenic gene program in BAT after 3 days of cold-exposure, however, *Atf4* expression in BAT was required for the cold-induced upregulation of *Fgf21* and both *Atf4* and *Ffg21* expression was required for cold-induced browning of white adipose tissue (WAT). Nonetheless, core body temperature was only significantly reduced in ATF4 BKO mice, but not in FGF21 BKO mice, when mice were cold exposed with free access to food. Noteworthy, in the absence food, both *Fgf21* and *Atf4* were required for thermoregulation in mice. Together, our study identified novel roles for ATF4 in BAT physiology in regulating core body temperature in a manner that is independent of *Fgf21* induction in thermogenic adipocytes or cold-induced browning. Our data points to an alternative mechanism downstream of *Atf4*, which may involve regulation of amino acid transport and metabolism^15^ in response to cold stress, that is required for cold-induced thermogenesis in mice.

## Results

### ATF4 and its downstream targets are induced in BAT after 3 days of cold exposure

**Figure 1:**
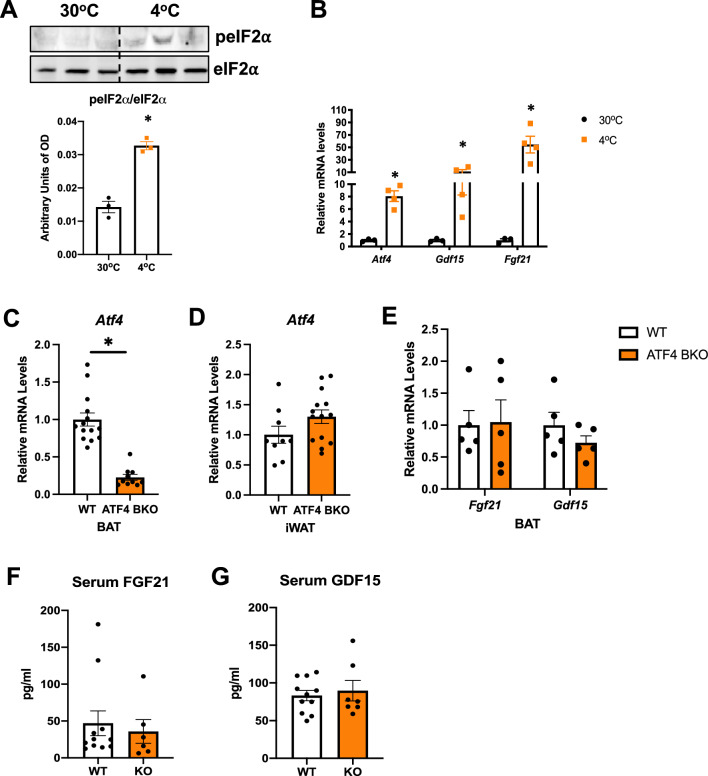
ATF4 and its downstream targets are induced in BAT after 3 days of cold exposure. (**A**) and (**B**) Data collected in BAT of WT mice exposed to 30 °C or 4 °C for 3 days. (**A**) Immunoblot of phosphorylated eIf2α normalized to total eIf2α and respective densitometric quantification. Images cropped from the same blot (n = 3). Optical density (OD). Original immunoblots are presented in Supplemental Figure S5. (**B**) mRNA expression of *Atf4*, *Fgf21* and *Gdf15* in BAT normalized to *Gapdh* (30 °C n = 3; 4 °C n = 4). (**C–G**) Data collected in WT and ATF4 BAT KO male and female mice at baseline conditions. (**C**) Relative mRNA expression of *Atf4* in BAT normalized to *Gapdh* (WT n = 14; KO n = 10). (**D**) Relative mRNA expression of *Atf4* in inguinal white adipose tissue (iWAT) normalized to *Gapdh* (WT n = 9; KO n = 14)*.* (**E**) Relative mRNA expression of *Fgf21* and *Gdf15* in BAT normalized to *Gapdh* (n = 5). (**F**) Serum FGF21 levels (WT n = 11; KO n = 6). (**G**) Serum GDF15 levels (WT n = 11; KO n = 7). Data are expressed as means ± SEM. Significant differences were determined by Student's *t*‐test, using a significance level of *P* < 0.05. *p < 0.05 significantly different vs. WT mice or 30 °C.

ATF4 was shown to be potently induced in BAT in response to acute cold-stress (4–6 h) following a 12-h fast, which correlated with high serum levels of FGF21 and growth differentiation factor 15 (GDF15), both downstream targets of ATF4^7^. We therefore sought to examine if this pathway was also induced after 3 days of cold exposure in *ad libitum*-fed conditions. Mice were acclimated to thermoneutrality (30 °C) for 7 days to attenuate brown adipose tissue thermogenic function, after which they were exposed to 4 °C for 3 days to induce thermogenesis. Our data demonstrated increased phosphorylation of the eukaryotic translation initiation factor 2A (eIF2α), which is upstream of ATF4 induction (Fig. 1A). Accordingly, mRNA levels of *Atf4*, and its downstream targets *Fgf21* and *Gdf15* were also induced in BAT (Fig. 1B) in response to 3 days of cold exposure. Three days of cold exposure has also been shown to induce browning of the inguinal white adipose tissue (iWAT)^16^. To determine if that was also the case in our model, we measured mRNA expression of thermogenic genes in iWAT of wild type (WT) mice cold exposed for 3 days, versus mice kept at 30 °C*.* Our data showed significant induction of thermogenic genes in iWAT of cold exposed mice (Supplemental Figure S1A), confirming cold-induced browning when mice are subjected to this cold-exposure protocol. *Atf4* mRNA expression was also induced in the inguinal white adipose tissue (iWAT) after 3 days of cold exposure, whereas *Fgf21* and *Gdf15* mRNA expression, although somewhat induced, were not statistically different (Supplemental Figure S1B). To investigate whether ATF4 induction in BAT is required for adaptive thermogenesis, we generated mice lacking ATF4 selectively in thermogenic adipocytes (ATF4 BKO mice). *Atf4* expression was significantly decreased in BAT of KO mice (Fig. 1C), while *Atf4* expression was preserved in the inguinal white adipose tissue (iWAT) (Fig. 1D) at room temperature conditions. *Atf4* expression was also measured in other tissues to confirm tissue specific deletion. No changes in *Atf4* mRNA levels were detected under baseline conditions between WT and ATF4 BKO mice in kidney, brain, mammary glands or in the eyes (Supplemental Figure S1C). *Fgf21* and *Gdf15* mRNA (Fig. 1E) expression in BAT and their respective circulating levels (Fig. 1F, G) were unchanged between WT and KO mice under baseline conditions (chow-fed mice at room temperature).

### ATF4 BAT KO mice have reduced BAT mass and mildly impaired glucose tolerance at baseline conditions

**Figure 2:**
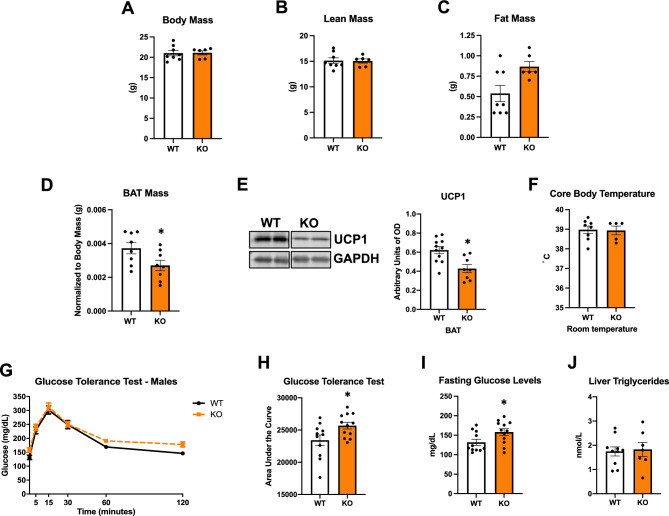
ATF4 BAT KO mice have reduced BAT mass and mildly impaired glucose tolerance at baseline conditions. Data collected under baseline conditions in ATF4 BKO (KO) male mice and their respective WT littermate controls. (**A**) Body mass. (**B**) Total lean mass. (**C**) Total fat mass*.* (WT n = 8; KO n = 6). (**D**) Brown adipose tissue (BAT) mass normalized to body mass (n = 8). (**E**) UCP1 protein levels in BAT normalized to GAPDH and respective densitometric quantification (WT n = 11; KO n = 8). Images cropped from the same blot. Optical density (OD). Original immunoblots are presented in Supplemental Figure S6. (**F**) Core body temperature measured with a rectal probe (WT n = 8; KO n = 5). (**G**) Glucose tolerance test (GTT). (**H**) Area under the curve for the GTT. (**I**) Fasting glucose levels (after a 6-h fast) (WT n = 11; KO n = 12). (**J**) Liver triglyceride levels (WT n = 10; KO n = 7). Data are expressed as means ± SEM. Significant differences were determined by Student's *t*‐test, using a significance level of *P* < 0.05. *p < 0.05 significantly different vs. WT mice.

Because global ATF4 deletion has been associated with changes in systemic metabolism^12,13^, we evaluated body composition, and glucose homeostasis in 8–10-week-old ATF4 BKO mice. Body mass (Fig. 2A) and total lean mass (Fig. 2B) were unchanged between KO mice and their WT littermate controls, while total fat mass was increased in KO mice (Fig. 2C). Although total fat mass was increased, brown adipose tissue (BAT) mass was modestly, but significantly reduced in ATF4 BKO mice (Fig. 2D and Supplemental Figure S2A), and iWAT and gonadal white adipose tissue (gWAT) mass were unchanged between genotypes (Supplemental Figure S2B and C). Interestingly, UCP1 protein levels were also significantly reduced in BAT of ATF4 BKO mice at baseline conditions (Fig. 2E), while rectal core body temperature was unchanged between genotypes (Fig. 2F). To assess the impact of *Atf4* deletion in thermogenic adipocytes on glucose homeostasis, we performed glucose tolerance tests (GTT). ATF4 BKO mice had mildly impaired glucose tolerance (Fig. 2G), as demonstrated by increased area under the curve for the GTT (Fig. 2H), and higher fasting glucose levels after a 6-h fast (Fig. 2I), while liver triglyceride levels were comparable between genotypes (Fig. 2J). Hence, under baseline conditions, ATF4 deletion in BAT has small effects in systemic glucose homeostasis. We believe that this mild impairment in glucose homeostasis could be related to the small increase in total fat mass observed in these animals.

### ATF4 expression in BAT is required for thermoregulation in mice

**Figure 3:**
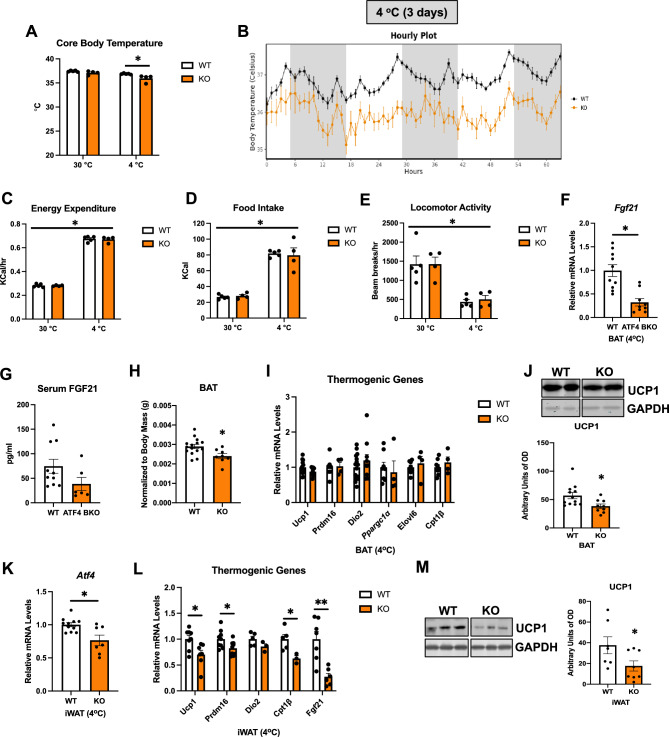
ATF4 expression in BAT is required for thermoregulation in mice. (**A–E**) Data collected in WT and ATF4 BKO (KO) male mice either during 3 days at thermoneutrality (30 °C) or during 3 days of cold exposure (4 °C). (**A**) Averaged core body temperature. (**B**) Core body temperature over time during cold exposure. (**C**) Energy expenditure. (**D**) Food intake. (**E**) Locomotor activity (WT n = 5; KO n = 4). (**F–M**) Data collected in WT and ATF4 BKO (KO) male mice after 3 days of cold exposure (4 °C). (**F**) Relative *Fgf21* mRNA expression in BAT normalized to *Gapdh* (WT n = 10; KO n = 9). (**G**) Serum FGF21 levels (WT n = 10; KO n = 6). (**H**) BAT mass normalized to body mass (WT n = 15; KO n = 8). (**I**) mRNA expression of thermogenic genes in BAT normalized to *Gapdh* (WT n = 8–14; KO n = 4–10). (**J**) Immunoblot of UCP1 in BAT normalized to GAPDH and its respective densitometric quantification. Images cropped from the same blot. Optical density (OD) (WT n = 12; KO n = 9). Original immunoblots are presented in Supplemental Figure S7A. (**K**) mRNA expression of *Atf4* in inguinal white adipose tissue (iWAT) normalized to *Gapdh* (WT n = 11; KO n = 7). (**L**) mRNA expression of thermogenic genes in iWAT normalized to *Gapdh* (WT n = 5–9; KO n = 3–8). (**M**) Immunoblot of UCP1 in inguinal white adipose tissue (iWAT) normalized to GAPDH and its respective densitometric quantification. Images cropped from the same blot (WT n = 7; KO n = 8). Optical density (OD). Original immunoblots are presented in Supplemental Figure S7B. Data are expressed as means ± SEM. Significant differences were determined by Student’s *t*‐test, using a significance level of *p* < 0.05. *Significantly different vs. WT mice or 30 °C.

To determine the requirement of ATF4 expression in BAT for adaptive thermogenesis, we monitored core body temperature and metabolic changes in ATF4 BKO mice and their WT littermate controls during 3 days at thermoneutrality (30 °C) and 3 days of cold exposure (4 °C). Although averaged core body temperature was unchanged between WT and KO mice at 30 °C, the averaged core body temperature during the 3 days of cold exposure was significantly reduced in KO mice (Fig. 3A, B). Despite having reduced thermoregulatory capacity, the metabolic phenotype of ATF4 BKO mice was largely unaffected, with no significant changes in energy expenditure (Fig. 3C), food intake (Fig. 3D), or locomotor activity (Fig. 3E) at thermoneutrality or during cold exposure, when compared to WT littermate controls (no effect associated with genotype). Mice were euthanized and tissues were harvested after the 3 days of cold exposure for molecular analysis. Cold-induced mRNA expression of *Fgf21* (Fig. 3F) was significantly reduced in ATF4 BKO mice. Although FGF21 serum levels trended to be lower in ATF4 BKO mice relative to their WT counterparts, no statistical differences were detected (Fig. 3G). BAT mass was slightly decreased in ATF4 BKO mice (Fig. 3H), however, relative mRNA expression of thermogenic genes was unchanged between genotypes (Fig. 3I). UCP1 protein levels (Fig. 3J) were modestly decreased in BAT of KO mice after cold exposure. Browning of WAT is induced in response to 3 days of cold exposure and is believed to contribute to thermoregulation in mice^16^. We, therefore, assessed browning of iWAT in WT and ATF4 BKO mice after 3 days of cold exposure. *Atf4* mRNA expression was significantly reduced in iWAT of KO mice after cold exposure (Fig. 3K). Moreover, activation of the thermogenic gene program was attenuated (Fig. 3L), and UCP1 protein levels were significantly reduced in iWAT of KO mice (Fig. 3M), suggesting impaired cold-induced browning. Together these data suggest that ATF4 expression in thermogenic adipocytes is required to support cold-induced thermogenesis in mice, and cold-induced browning in iWAT. Furthermore, ATF4 also regulates *Fgf21* mRNA expression in thermogenic adipocytes in response to cold. However, whether FGF21 induction in thermogenic adipocytes is required to mediate these ATF4-dependent changes to regulate thermogenesis remained unresolved.

### FGF21 BAT KO mice have normal body weight and glucose homeostasis at baseline conditions

**Figure 4:**
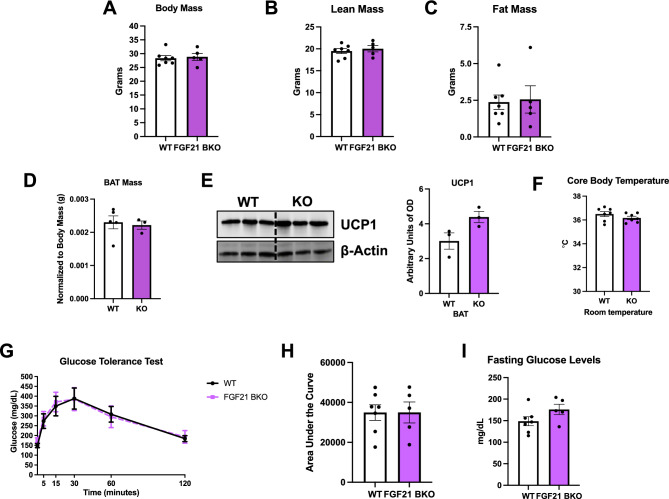
FGF21 BAT KO mice have normal body weight and glucose homeostasis at baseline conditions. Data collected under baseline conditions in FGF21 BKO (KO) male mice and their respective WT littermate controls. (**A**) Body mass. (**B**) Total lean mass. (**C**) Total fat mass (WT n = 7; KO n = 5). (**D**) Brown adipose tissue (BAT) mass normalized to body mass (WT n = 5; KO n = 3). (**E**) UCP1 protein levels in BAT normalized to β-Actin and respective densitometric quantification (n = 3). Optical density (OD). Original immunoblots are presented in Supplemental Figure S8. (**F**) Core body temperature measured with a rectal probe (n = 7). (**G**) Glucose tolerance test (GTT). (**H**) Area under the curve for the GTT. (**I**) Fasting glucose levels (after a 6-h fast) (WT n = 7; KO n = 5). Data are expressed as means ± SEM. Significant differences were determined by Student's *t*‐test, using a significance level of *P* < 0.05.

To test the role of *Fgf21* expression in thermogenic adipocytes for adaptive thermogenesis, we selectively deleted *Fgf21* in *Ucp1*-expressing adipocytes (FGF21 BKO). Because global FGF21 deletion has been associated with changes in glucose and energy homeostasis^17,18^, we evaluated body composition, and glucose homeostasis in 15-week-old FGF21 BKO mice fed chow diet. Body mass (Fig. 4A), total lean mass (Fig. 4B) and total fat mass (Fig. 4C) were unchanged between KO mice and their WT littermate controls. BAT weight normalized to body weight (Fig. 4D), UCP1 protein levels in BAT (Fig. 4E) and core body temperature (Fig. 4F) were also similar between WT and KO mice under baseline conditions. To assess the impact of FGF21 deletion in thermogenic adipocytes on glucose homeostasis, we performed glucose tolerance tests (GTT). FGF21 BKO mice had similar glucose clearance as WT mice, as demonstrated by unchanged areas under the curve for the GTT (Fig. 4G, H), and comparable fasting glucose levels after a 6-h fast (Fig. 4I).

### FGF21 expression in thermogenic adipocytes is dispensable for thermoregulation

**Figure 5:**
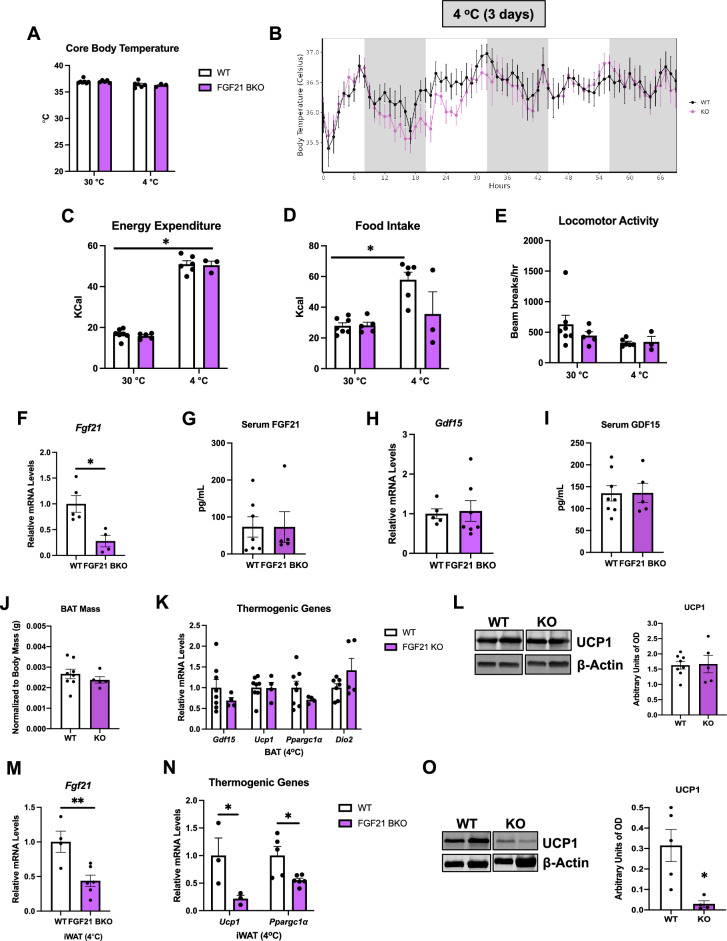
FGF21 Expression in Thermogenic Adipocytes Is Dispensable for Thermoregulation. (**A–E**) Data collected in WT and FGF21 BKO (KO) male mice either during 3 days at thermoneutrality (30 °C) or during 3 days of cold exposure (4 °C). (**A**) Averaged core body temperature. (**B**) Core body temperature over time during cold exposure. (**C**) Energy expenditure. (**D**) Food intake. (**E**) Locomotor activity (WT n = 5–7; KO n = 3–5). (**F–O**) Data collected in WT and FGF21 BKO (KO) male mice after 3 days of cold exposure (4 °C). (**F**) Relative *Fgf21* mRNA expression in BAT normalized to *Tbp* (tata box protein) (WT n = 5; KO n = 6). (**G**) Serum FGF21 levels (WT n = 7; KO n = 5). (**H**) Relative *Gdf15* mRNA expression in BAT normalized to *Tbp* (WT n = 5; KO n = 7). (**I**) Serum GDF15 levels (WT n = 8; KO n = 5). (**J**) BAT mass normalized to body mass (WT n = 8; KO n = 5). (**K**) mRNA expression of thermogenic genes in BAT normalized to *Tbp* (WT n = 7–8; KO n = 4–5). (**L**) Immunoblot of UCP1 in BAT normalized to β-Actin and its respective densitometric quantification (WT n = 8; KO n = 5). Images cropped from the same blot. Optical density (OD). Original immunoblots are presented in Supplemental Figure S9A. (**M**) mRNA expression of *Fgf21* in inguinal white adipose tissue (iWAT) normalized to *Tbp* (WT n = 4; KO n = 6). (**N**) mRNA expression of thermogenic genes in iWAT normalized to *Tbp* (WT n = 3–5; KO n = 3–6). (**O**) Immunoblot of UCP1 in inguinal white adipose tissue (iWAT) normalized to β-Actin and its respective densitometric quantification. Images cropped from the same blot (WT n = 5; KO n = 4). Optical density (OD). Original immunoblots are presented in Supplemental Figure S9B. Data are expressed as means ± SEM. Significant differences were determined by Student's *t*‐test, using a significance level of *p* < 0.05. *Significantly different vs. WT mice or 30 °C.

*Fgf21* mRNA expression was significantly reduced in ATF4 BKO mice after cold exposure. Therefore, we directly tested the role of cold-induced *Fgf21* expression in thermogenic adipocytes for adaptive thermogenesis by exposing FGF21 BKO mice to 4 °C for 3 days. We monitored core body temperature and metabolic changes in FGF21 BKO mice and their WT littermate controls for 3 days at thermoneutrality (30 °C) and 3 days of cold exposure (4 °C). Averaged core body temperature, as measured by telemetry, was unchanged between WT and FGF21 BKO mice at thermoneutrality (Fig. 5A) or during the 3 days of cold exposure (Fig. 5A, B). Of note, 1 WT mouse and 2 KO mice died unexpectedly soon after the temperature was changed to 4 °C and were removed from the analysis. Regarding energy homeostasis, energy expenditure (Fig. 5C) and locomotor activity (Fig. 5E) were unaffected in KO mice either at 30 °C or 4 °C. Food intake was significantly increased in WT mice after cold exposure, which was attenuated in KO mice (Fig. 5D). Although *Fgf21* mRNA levels were significantly reduced in BAT (Fig. 5F), FGF21 serum levels (Fig. 5G) were unchanged between genotypes, indicating that BAT-derived FGF21 does not contribute to FGF21 circulating levels after 3 days of cold exposure. To determine whether compensatory changes in GDF15 levels could have affected food intake in FGF21 BKO mice, we measured GDF15 levels in BAT and in the serum after 3 days of cold exposure, but detected no differences in *Gdf15* mRNA expression (Fig. 5H) or circulating levels (Fig. 5I) between genotypes. BAT mass normalized to body weight was unaffected by FGF21 deletion (Fig. 5J). Likewise, expression of thermogenic genes (Fig. 5K), and UCP1 protein levels were similar in BAT of WT and FGF21 BKO mice (Fig. 5L). Next, we evaluated the effects of FGF21 deletion on cold-induced browning of iWAT. *Fgf21* mRNA levels were significantly reduced in iWAT of cold-exposed FGF21 BKO mice (Fig. 5M), which correlated with reduced mRNA levels of the thermogenic genes *Ucp1* and *Ppargc1α* (Fig. 5N), and with significantly lower UCP1 protein levels (Fig. 5O). Together, our data reinforce a role for FGF21 on cold-induced browning of WAT and suggest that impaired iWAT browning, when BAT thermogenesis is intact, is insufficient to hamper thermoregulation in mice, at least under the conditions tested in this study. Our data also indicate that defective *Fgf21* expression in ATF4 BKO mice is likely dispensable for the impaired thermoregulatory capacity in mice fed *ad-libitum*. Finally, our data suggest that FGF21 induction in BAT does not contribute to FGF21 serum levels in response to 3 days of cold exposure, and it is dispensable for cold-induced thermogenesis in *ad libitum*-fed mice. The possibility remains that BAT-derived FGF21 as well as browning of iWAT could be required to sustain thermogenesis in the context of prolonged cold exposure (one week or longer), which was not tested in the present study.

### Transcriptome analysis reveals downregulation of genes involved in amino acid metabolism in ATF4 BKO mice

**Figure 6:**
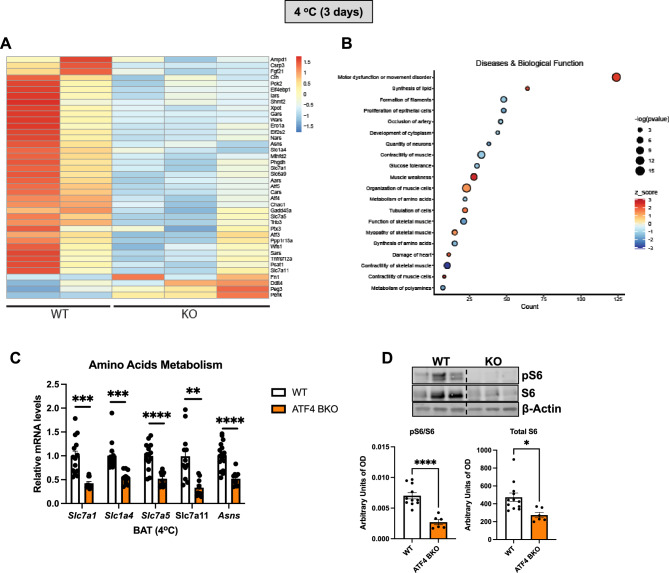
Transcriptome Analysis Reveals Downregulation of Genes Involved in Amino Acid Metabolism in ATF4 BKO Mice. (**A, B**) Data collected from brown adipose tissue (BAT) of WT and ATF4 BKO mice cold exposed for 3 days (Differential gene expression and Ingenuity Pathway Analysis). (**A**) Heatmap of differentially expressed genes annotated in the IPA database as ATF4 targets. Plotted are log2(x + 1) transformed counts scaled by row. (**B**) Differentially expressed genes (DEGs) in ATF4KO BAT have a significant overlap (adjusted *p*-value ≤ 0.05) with genes that are annotated to be involved in various diseases or with genes that share common biological functions (adjusted *p*-value ≤ 0.05). The number of genes in the data set annotated as being involved in the disease or have a particular biological function is plotted on the x-axis. Bubble size = -log (adjusted *p*-value) and color = z-score; indicates the potential impact of the differential gene expression pattern on the disease or biological function. (**A**) Relative mRNA expression of amino acid transporter and metabolism genes in BAT of ATF4 BKO mice normalized to *Tbp* expression (WT n = 10–14; KO n = 6–10). (**D**) Immunoblot of phosphorylated S6 in BAT normalized to total S6 and total S6 normalized to β-Actin and their respective densitometric quantification (WT n = 11; KO n = 6). Images cropped from the same blot. Optical density (OD). Original immunoblots are presented in Supplemental Figure S10. Data are expressed as means ± SEM. Significant differences were determined by Student's *t*‐test, using a significance level of *p* < 0.05. *p<0.05; **p < 0.01; ***p < 0.001; ****p < 0.0001 significantly different vs. WT mice.

To gain insight into the molecular mechanisms driving impaired thermoregulation in ATF4 BKO mice, we performed RNA sequencing (RNASeq) in BAT collected from 10 to 12-week-old mice lacking ATF4 in thermogenic adipocytes (ATF4 BAT KO) and their respective wild-type (WT) littermate controls after 3 days of cold exposure. Principal component analysis showed separation between WT and ATF4 BKO mice. Despite high variance within the WT group, ATF4 BKO mice clustered well (Supplemental Figure S3A). In addition to confirming downregulation of *Fgf21* in ATF4 BKO mice, our transcriptome data revealed that several amino acid transporters were amongst the top downregulated genes in ATF4-deficient BAT (Fig. 6A). Furthermore, Ingenuity Pathway Analysis uncovered that genes involved in amino acid synthesis and metabolism were repressed in cold-exposed ATF4 BKO mice relative to their WT counterparts (Fig. 6B). Indeed, a role for ATF4 in regulating amino acid transport and metabolism has been previously reported^15^. Repression of amino acid metabolism-related genes in ATF4 BKO mice after 3 days of cold exposure was confirmed by qPCR (Fig. 6C); Meanwhile, these genes were unchanged in BAT of cold-exposed FGF21 BAT KO mice under the same conditions (Supplemental Fig. S3B). Of note, defective branched-chain amino acids (BCAA) uptake and oxidation in BAT leads to reduced thermogenesis in mice^19^, suggesting that BCAA catabolism in BAT is required for proper thermoregulation. Furthermore, a recent study demonstrated that ATF4 overexpression in BAT improves cold-induced thermogenesis, which seems to be dependent on activation of mTOR signaling^8^. Our data shows that mTOR signaling pathway, as measured by total and phosphorylated ribosomal protein S6, was reduced in BAT of cold-exposed ATF4 BKO mice relative to WT controls (Fig. 6D). Together, these data suggest that ATF4, but not FGF21 is required to regulate amino acid import and metabolism in BAT during cold exposure in *ad libitum*-fed mice and may also mediate mTOR signaling in response to cold, which could contribute to supporting thermoregulation in mice.

### ATF4 and FGF21 expression in thermogenic adipocytes are required to maintain core body temperature in cold-exposed mice under fasting conditions

**Figure 7:**
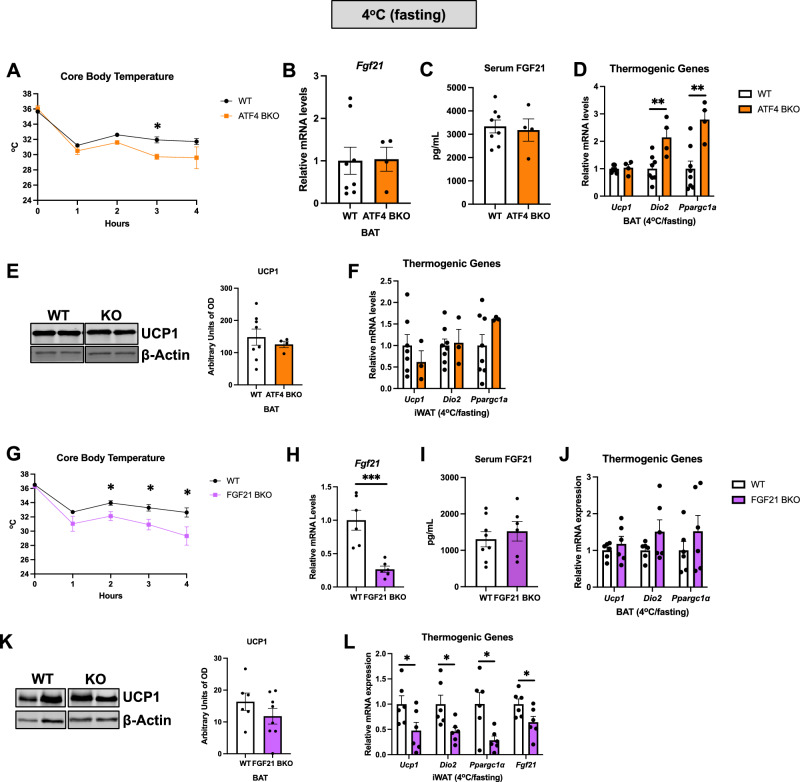
ATF4 and FGF21 Expression in Thermogenic Adipocytes Is Required to Maintain Core Body Temperature in Mice Cold Exposed Under Fasting Conditions. (**A–F**) Data collected in WT and ATF4 BKO mice cold exposed for 4 h following a 12-h fast. (**A**) Hourly core body temperature. (**B**) *Fgf21* mRNA levels in BAT of ATF4 BKO mice normalized to *Tbp* (WT n = 8; KO n = 4). (**C**) FGF21 serum levels (WT n = 8; KO n = 4). (**D**) mRNA expression of thermogenic genes in BAT normalized to *Tbp* (WT n = 8; KO n = 4). (**E**) Immunoblot of UCP1 in BAT normalized to β-actin and its respective densitometric quantification (WT n = 8; KO n = 4). Images cropped from the same blot. Optical density (OD). Original immunoblots are presented in Supplemental Figure S11A. (**F**) mRNA expression of thermogenic genes in iWAT normalized to *Tbp* (WT n = 8; KO n = 4). (**G–L**) Data collected in WT and FGF21 BKO mice cold exposed for 4 h following a 12-h fast. (**G**) Hourly core body temperature (WT n = 8; KO n = 6). (**H**) *Fgf21* mRNA levels in normalized to *Tbp* (WT n = 6; KO n = 6)*.* (**I**) FGF21 serum levels (WT n = 8; KO n = 6). (**J**) mRNA expression of thermogenic genes in BAT normalized to *Tbp* (WT n = 8; KO n = 6). (**K**) Immunoblot of UCP1 in BAT normalized to β-actin and its respective densitometric quantification (WT n = 6; KO n = 7). Images cropped from the same blot. Optical density (OD). Original immunoblots are presented in Supplemental Figure S11B. (**L**) mRNA expression of thermogenic genes in iWAT normalized to *Tbp* (WT n = 6; KO n = 6). Data are expressed as means ± SEM. Significant differences were determined by Student's *t*‐test, using a significance level of *p* < 0.05. *p < 0.05; **p < 0.01; ***p < 0.001 significantly different vs. WT mice.

An earlier study reporting potent activation of ATF4 in BAT in response to acute cold exposure was performed following fasting, which correlated with increased FGF21 serum levels^7^. To test the role of ATF4 in thermoregulation and in FGF21 induction in BAT under these conditions, a separate cohort of ATF4 BKO mice was cold-exposed for 4 h following a 12-h fast. Core body temperature was significantly reduced to a greater extent in ATF4 BKO relative to their WT counterparts (Fig. 7A). In contrast to *ad libitum*-fed mice, under fasting conditions, FGF21 expression in BAT was not reduced (Fig. 7B) nor were FGF21 serum levels changed (Fig. 7C). These data suggest that additional ATF4-independent mechanisms exist that regulate FGF21 in BAT during acute cold exposure following fasting. Our data support prior reports that FGF21 secretion from liver is likely the primary contributor to FGF21 serum levels during fasting and acute cold exposure^20,21^. Unexpectedly, a subset of thermogenic genes was significantly induced in BAT of ATF4 BKO mice (Fig. 7D), while UCP1 protein levels were unchanged between ATF4 BKO mice and WT control mice in BAT (Fig. 7E), indicating that additional ATF4-independent mechanisms maintain normal UCP1-dependent thermogenesis under these conditions of combined nutritional and cold stress. Moreover, expression of thermogenic genes in iWAT that were previously repressed in cold-exposed *ad libitum*-fed ATF4 BKO mice were similar between WT and ATF4 BKO mice under simultaneous nutritional and cold stress (Fig. 7F). Likewise, expression of AA transporters, that were previously repressed, was similar in BAT of ATF4 BKO and WT controls (Supplemental Fig. S4). Taken together, these data indicate that under fasting conditions additional ATF4-independent mechanisms are activated to sustain thermogenic function. Although FGF21 BKO mice were able to properly thermoregulate when cold exposed under ad libitum-fed conditions, in the absence of food they had reduced tolerance to cold relative to their WT counterparts (Fig. 7G). *Fgf21* mRNA levels were significantly reduced in BAT (Fig. 7H), but FGF21 serum levels (Fig. 7I) were similar between genotypes. Thermogenic gene expression (Fig. 7J) and UCP1 protein levels (Fig. 7K) in BAT were also unchanged between FGF21 BKO mice and their WT controls. Cold-induced activation of thermogenic genes in iWAT was also reduced in FGF21 BKO mice under fasting conditions (Fig. 7L). Together, these data suggest that ATF4 and FGF21 expression in BAT may independently regulate core body temperature in mice during acute cold exposure following fasting via yet unidentified mechanisms, despite adaptive mechanisms that maintain markers of BAT thermogenic activity.

## Discussion

ATF4 was recently shown to be potently induced in BAT in response to acute cold exposure, which correlated with increased FGF21 serum levels^7^. Intriguingly, mice with global deletion of the *Atf4* gene have increased energy expenditure and induced *Ucp1* expression in BAT^13^. Furthermore, these mice are better able to maintain core body temperature during a 7-h cold challenge^14^. Nonetheless, a recent study demonstrated that these effects are largely centrally mediated rather than a result of *Atf4* deficiency in BAT, as adult-onset conditional *Atf4* deletion in agouti-related peptide neurons in the hypothalamus of mice is sufficient to increase energy expenditure and enhance thermogenesis in BAT^22^. Indeed, our recently published study demonstrated that *Atf4* induction in BAT resulting from mitochondrial stress is required for the induction and secretion of FGF21 as a batokine and to improve thermoregulation in mice. In this model, mice with concomitant deletion of the mitochondrial protein OPA1 and either ATF4 or FGF21 specifically in thermogenic adipocytes had impaired thermoregulatory capacity in response to 3 days of cold exposure (4 ºC)^9^. Moreover, a recent study showed that ATF4 overexpression in BAT improves cold-induced thermogenesis in mice^8^. In the aforementioned studies, *Atf4* was induced in BAT via genetic manipulation using a constitutive *Ucp1*-Cre model, which could have led to additional adaptations over time. However, whether physiological induction of endogenous ATF4 in BAT in response to cold is required to support thermoregulation in mice was not known. In the present study, we hypothesized that activation of ATF4 in response to cold is required for proper thermoregulation in mice and to induce FGF21 as a batokine.

Thermogenic stimulation has been shown to strongly induce *Fgf21* expression in BAT via mechanisms that involve canonical activation of the β-adrenergic signaling pathway^16^. Our recent study demonstrated that FGF21 can be induced when BAT’s thermogenic function is impaired, via ATF4 activation^9^. ATF4 binds to the FGF21 promoter and induces its transcription in several cell models^23–25^. However, whether ATF4 is required for cold-induced upregulation of FGF21 in BAT remained to be elucidated. In the present study, we show that mice lacking ATF4 specifically in thermogenic adipocytes (ATF4 BKO) have reduced core body temperature after 3 days of cold exposure in the absence of major changes in the expression of thermogenic genes in BAT. Additionally, energy expenditure was unchanged in these animals at thermoneutrality and during cold exposure. Although thermogenic gene activation was maintained in BAT, cold-induced *Fgf21* mRNA expression was reduced in thermogenic adipocytes (BAT and iWAT), and cold-induced browning of WAT was attenuated in ATF4 BKO mice. Thus, ATF4 induction is required for increasing *Fgf21* expression in response to short-term cold stress in BAT and iWAT under *ad-libitum* fed conditions. Therefore, we directly tested whether FGF21 is required to promote cold-induced thermogenesis, in mice lacking FGF21 selectively in thermogenic adipocytes.

Cold exposure stimulates FGF21 expression in both humans and animal models. However, the source of circulating FGF21 and its metabolic consequences for adaptive thermogenesis are still incompletely understood^26,27^. A study in FGF21 global KO mice showed that ablation of FGF21 leads to an impaired response to cold stress when mice adapted at 27 °C were transferred to 5 °C for 3 days^16^. In UCP1 knockout (KO) mice, BAT was suggested as a potential source of circulating FGF21 following long-term cold exposure, which was associated with increased browning of subcutaneous WAT relative to WT mice. However, a recent study in UCP1-FGF21 double knockout mice demonstrated that FGF21 is dispensable for this phenotype, including the changes in energy expenditure and thermoregulation during prolonged cold exposure in the UCP1 KO model^28^. Furthermore, a recent study demonstrated that liver-derived, but not adipose-derived FGF21 is required to maintain core body temperature in the first few hours of cold exposure^20^. Therefore, although FGF21 may be dispensable for the adaptation to gradual long-term cold exposure in UCP1 KO mice, liver derived FGF21 is likely required for thermoregulation in more acute scenarios. Although we observed no changes in FGF21 serum levels after 3 days of cold exposure, our data in ATF4 BKO mice, in which cold-induced *Fgf21* expression is diminished in BAT and WAT, suggest that FGF21 expression in thermogenic adipocytes might be necessary for the regulation of core body temperature in response to short-term cold exposure in mice, potentially via autocrine/paracrine actions. However, our data in FGF21 BKO mice revealed no changes in BAT thermogenic activation or core body temperature after 3 days of cold exposure when mice have free access to food, even though cold-induced browning of WAT was attenuated. These data reinforce a role for FGF21 on cold-induced browning of WAT, as previously reported^16,29,30^, and suggest that impaired browning is insufficient to hamper effective thermoregulation in mice when BAT thermogenesis is preserved. Our data also indicate that defective *Fgf21* expression in ATF4 BKO mice is unlikely to play a role on the impaired thermoregulatory capacity observed in these mice under fed conditions, at least during short-term cold exposure. Whether FGF21 expression in thermogenic adipocytes and FGF21-induced browning of WAT are required to maintain thermogenesis during prolonged cold exposure remains to be determined.

Transcriptome data in BAT of cold-exposed ATF4 BKO mice revealed that, in addition to reduced *Fgf21* transcript levels, gene expression of several amino acid transporters, including of branched-chain amino acids (BCAA), and amino acid metabolism genes was repressed relative to WT control mice. ATF4 has been previously shown to regulate amino acid transport and metabolism through mTORC1 mediated mechanisms in cell models^15^. A recent study showed that cold stimuli potently increase mitochondrial BCAA uptake and oxidation in BAT, leading to enhanced BCAA clearance in the circulation. Furthermore, defective BCAA catabolism in BAT impaired BCAA clearance and reduced thermogenesis in mice^19^, suggesting that BCAA catabolism in BAT is required to support thermogenesis. Indeed, a recently published study in mice lacking the ISR kinase GCN2, which is induced in response to amino acid deprivation, demonstrated that GCN2 directs ISR-driven increases in amino acid transport into BAT contributing to the maintenance of core body temperature during cold stress^31^. Furthermore, over the last several years, the mechanistic target of rapamycin (mTOR) signaling pathway has been reported to affect BAT function in rodents. Studies from independent groups showed that mTORC1 activity is highly induced by acute and chronic cold exposure and β3-adrenergic receptor stimulation^32–34^. Loss of mTORC1 in adipocytes reduces BAT size and completely prevents cold-induced BAT expansion, mitochondrial biogenesis, and oxidative metabolism in mice^33–35^. Here, we show that phosphorylation of the downstream mTOR target ribosomal protein 6 (S6) is reduced in ATF4-deficient BAT after 3 days of cold exposure, suggesting that ATF4 induction in response to cold may regulate mTOR-dependent pathways to promote thermogenesis in BAT. Moreover, a recent study showed that ATF4 overexpression in BAT improves cold-induced thermogenesis, which seems to be dependent on increased S6 phosphorylation, and is attenuated upon treatment with the mTOR inhibitor rapamycin^8^. Although not directly tested in the present study, our data suggest that ATF4 induction in BAT is required to regulate cold-induced amino acid transport and metabolism to support thermogenesis in ad libitum fed mice.

In the present study we reveal a novel role for FGF21 expression in thermogenic adipocytes in the modulation of core body temperature under fasting conditions. Although FGF21 BKO mice showed no change in core body temperature when cold exposed under *ad libitum*-fed conditions, acute cold exposure following an overnight fast reduced core body temperature in FGF21 BKO mice to a similar degree as observed in ATF4 BKO mice. Under these conditions, ATF4 was dispensable for regulating *Fgf21* and amino acid transporters in BAT suggesting that additional ATF4-independent mechanisms are recruited under conditions of combined dietary and cold stress. FGF21 serum levels were similar in both ATF4 and FGF21 BKO mice relative to their WT counterparts, consistent with liver being the predominant source of circulating FGF21 under these conditions, as previously shown^20,21^. Additional studies will be needed to clarify the molecular mechanisms underlying changes in core body temperature, which we show occur independently of changes in UCP1 protein levels in BAT of ATF4 BKO or FGF21 BKO mice. Furthermore, although browning of iWAT was unaffected in ATF4 BKO mice, FGF21 BKO had reduced expression of thermogenic genes in iWAT, suggesting attenuated browning, via FGF21-dependent mechanisms.

In conclusion, by using mouse models of selective deletion of *Atf4* and *Fgf21* in thermogenic adipocytes, we identify overlapping and non-overlapping roles for ATF4 and FGF21 in thermoregulation in mice. Together, our data demonstrated that *Atf4* deletion in thermogenic adipocytes impairs thermoregulation in mice, in a manner that although potentially dependent of ATF4-mediated *Fgf21* expression or browning of WAT in *ad libitum*-fed mice, is independent of FGF21 under conditions of more severe combined nutritional and cold stress. ATF4 deficiency also reduces expression of amino acid transporter genes and impairs mTOR signaling, suggesting ATF4 might contribute to BAT thermogenesis by increasing amino acid metabolism and by activating mTOR signaling in response to cold exposure. In contrast, *ad libitum*-fed FGF21 BKO mice had preserved thermoregulation, and normal activation of thermogenic genes in BAT, despite having reduced browning of WAT. In the absence of food, both ATF4 and FGF21 are independently required to maintain core body temperature in mice regardless of changes in UCP1 levels. Future studies will focus on identifying the mechanisms downstream of ATF4 and FGF21 that regulate core body temperature under fasting conditions and elucidating whether increasing amino acid uptake and catabolism in BAT is sufficient to maintain core body temperature in ATF4 BKO mice.

## Research design and methods

### Animals and animal care

Experiments were performed in male mice on a C57Bl/6J background, unless otherwise stated. ATF4^fl/fl^^36^ and FGF21^fl/fl^ mice^18^ were generated as previously described. Transgenic mice expressing cre recombinase under the control of the *Ucp1* promoter (Tg (Ucp1-cre)1Evdr)^37^ were acquired from the Jackson Laboratories (#024670), and were crossed with ATF4^fl/fl^ and FGF21^fl/fl^ mice to promote selective deletion of these genes in thermogenic adipocytes (ATF4 BKO and FGF21 BKO mice, respectively). ATF4^fl/fl^ and FGF21^fl/fl^ mice not expressing the Cre recombinase were used as wild type (WT) controls.

Mice were weaned at 3 weeks of age and kept on standard chow (2920X Harlan Teklad, Indianapolis, IN, USA). Animals were housed at 22 °C with a 12‐h light, 12‐h dark cycle with free access to water and standard chow, unless otherwise noted. For cold exposure experiments, mice were acclimated to 30 °C (thermoneutral temperature for mice) for 7 days to dampen brown adipose tissue thermogenesis prior to being cold-exposed (4 °C). All mouse experiments presented in this study were conducted in accordance with animal research guidelines from the National Institutes of Health (NIH) and were approved by the University of Iowa Institutional Animal Care and Use Committee (IACUC) (protocol #: 3032294). The study described herein are reported in accordance with ARRIVE guidelines.

### Cold exposure experiments

Core body temperature telemeters (Respironics, G2 E-Mitter, Murrysville, PA, USA) were surgically implanted into the abdominal cavity of 8–10-week-old mice. The mice were then allowed to recover for 6 days post-surgery, while individually housed in a rodent environmental chamber (Power Scientific, Pipersville, PA, USA) at 30 °C. Mice were then transferred to an OxyMax Comprehensive Lab Animal Monitoring System (CLAMS, Columbus Instruments International) and singly housed at 30 °C for 3 days, followed by 4 °C for 3 days , as previously described^16^. Core body temperature was recorded every 17 min throughout the experiment, along with O_2_ and CO_2_ levels, food intake, and ambulatory activity, as estimated by photoelectric beam breaks in the X + Y plane. Mice were fed ad libitum throughout the study. A separate cohort of mice underwent acute cold exposure following fasting. For these studies, 12-week-old mice were initially individually housed in the rodent environmental chamber at 30 °C for 7 days. At the end of the seventh day, mice were fasted for 12 h (7 pm–7 am). The initial temperature (t0) was recorded using a rectal probe (Fisher Scientific, Lenexa, KS, USA) at 7 am on day 8, after which the temperature was switched to 4 °C. Once the desired temperature was reached, we recorded rectal temperatures hourly for up to 4 h during cold exposure. Mice remained without food for the entirety of the experiment.

### Glucose tolerance tests, nuclear magnetic resonance, and serum analysis

Glucose tolerance tests (GTT) and measurements of fasting glucose levels were performed as previously described^9^. Serum FGF21 (BioVendor ELISA kit, Asheville, NC, USA) and GDF15 (R&D Systems, Minneapolis, MN, USA) were measured using commercially available kits according to the manufacturers’ directions. Whole body composition was measured by nuclear magnetic resonance in the Bruker Minispec NF‐50 instrument (Bruker, Billerica, MA, USA)^9^.

### Analysis of triglyceride levels

Triglycerides levels were measured in the liver of ATF4 BKO mice, using the EnzyChrom™ Triglyceride Assay Kit (BioAssay Systems, Hayward, CA, USA), as previously described^9^.

### RNA extraction and quantitative RT–PCR

RNA concentration was determined by measuring the absorbance at 260 and 280 nm using a spectrophotometer (NanoDrop 1000, NanoDrop products, Wilmington, DE, USA). Total RNA (1 μg) was reverse‐transcribed using the High‐Capacity cDNA Reverse Transcription Kit (Applied Biosystems, Waltham, MA, USA), followed by qPCR reactions using SYBR Green (Life Technologies, Carlsbad, CA, USA)^38^. Samples were loaded in a 384‐well plate in triplicate, and real‐time polymerase chain reaction was performed with an ABI Prism 7900HT instrument (Applied Biosystems, Waltham, MA, USA). The following cycle profile was used: 1 cycle at 95 °C for 10 min; 40 cycles of 95 °C for 15 s; 59 °C for 15 s, 72 °C for 30 s, and 78 °C for 10 s; 1 cycle of 95 °C for 15 s; 1 cycle of 60 °C for 15 s; and 1 cycle of 95 °C for 15 s. Data were calculated using the delta-delta Ct method and normalized to either *Gapdh* or *Tbp* expression. Results are shown as relative mRNA levels. qPCR primers were designed using Primer‐Blast or previously published sequences^39^. Utilized primers are listed in Table 1.

**Table 1:** Primer sequences.

Gene name	Forward	Reverse
*Fgf21*	TGACGACCAAGACACTGAAGC	TTTGAGCTCCAGGAGACTTTCTG
*Atf4*	AGCAAAACAAGACAGCAGCC	ACTCTCTTCTTCCCCCTTGC
*Ucp1*	GTGAAGGTCAGAATGCAAGC	AGGGCCCCCTTCATGAGGTC
*Prdm16*	CAGCACGGTGAAGCCATTC	GCGTGCATCCGCTTGTG
*Gapdh*	AACGACCCCTTCATTGAC	TCCACGACATACTCAGCAC
*Dio2*	AATTATGCCTCGGAGAAGACCG	GGCAGTTGCCTAGTGAAAGGT
*Cpt1b*	TGCCTTTACATCGTCTCCAA	AGACCCCGTAGCCATCATC
*Evlov6*	TCAGCAAAGCACCCGAAC	AGCGACCATGTCTTTGTAGGAG
*Ppargc1α*	GTAAATCTGCGGGATGATGG	AGCAGGGTCAAAATCGTCTG
*Gdf15*	GAGAGGACTCGAACTCAGAAC	GACCCCAATCTCACCTCTG
*Slc7a1*	CGT GAG TAC GCG ATC CTT GT	AGG ACC AAG ATG GAC TCG GA
*Slc1a4*	GTG GCA TCG CTG TTG CTT AC	GAC GTA GTG AAT GCG GCA AC
*Slc7a11*	GTC TGC CTG TGG AGT ACT GT	ATT ACG AGC AGT TCC ACC CA
*Slc7a5*	ATC GTA GGT CCT GCC ATG TG	ACC GTG TCT GAG CTA GTT GC
*Asns*	TAC AAC CAC AAG GCG CTA CA	AAG GGC CTG ACT CCA TAG GT
*Tbp*	ACC CTT CAC CAA TGA CTC CTA TG	TGA CTG CAG CAA ATC GCT TGG

### RNA sequencing

Sequencing libraries were prepared using the Illumina TruSeq mRNA Stranded kit and sequenced on a HiSeq4000. Two approaches were used for read alignment, mapping, and quantification. First, a workflow using HISAT2 (v2.1.0), featureCounts (v1.6.3), and DESeq2 (v1.22.2) was performed^40–42^. The second approach used pseudo-alignment and quantification with Kallisto (v0.45.0) and DESeq2 for differential expression analysis^43^. Ingenuity® Pathway Analysis (IPA®) software from Qiagen was utilized for identification of potentially modified pathways. Data visualization was performed using pheatmap and ggplot2 packages in R. RNA-seq data have been deposited to the GEO database under the accession number GSE227060.

### Western blot analysis

Immunoblotting analysis was performed as previously described^44^. Approximately, 50 mg of frozen tissue was homogenized in 200 μl lysis buffer containing (in mmol/l) 50 HEPES, 150 NaCl, 10% glycerol, 1% Triton X‐100, 1.5 MgCl_2_, 1 EGTA, 10 sodium pyrophosphate, 100 sodium fluoride, and 100 μmol/l sodium vanadate. Right before use, HALT protease/phosphatase inhibitors (Thermo Fisher Scientific, Waltham, MA, USA) were added to the lysis buffer and samples were processed using the TissueLyser II (Qiagen Inc., Germantown, MD, USA). Tissue lysates were resolved on SDS–PAGE and transferred to nitrocellulose membranes (Millipore Corp., Billerica, MA, USA). Membranes were incubated with primary antibodies overnight at 4 °C and with secondary antibodies for 1 h, at room temperature. The data was analyzed using Image Studio Lite (LI-COR Technologies, Lincoln. NE, USA) and was normalized by the specified loading controls. Data is represented as arbitrary units of optical density (OD). Immunoblot membranes were cut prior to hybridization with antibodies.

### Antibodies

Primary Antibodies: GAPDH (1:1,000, Cell Signaling Technology, Danvers, MA, USA, #2118), UCP1 (1:1,000, Abcam, Boston, MA, USA, #Ab10983), phosphorylated eIF2α serine 51 (1:1,000, Cell Signaling Technology, #3597), total eIF2α (1:500, Santa Cruz Biotechnology, Dallas, TX, USA, #SC81261), phosphorylated S6 (1:1,000, Cell Signaling Technology, #4858), total S6 (1:1,000, Cell Signaling Technology, #2317) and β-actin (1:1,000, Sigma Aldrich, St. Louis, MO, USA, # A2066). Secondary antibodies: IRDye 800CW anti‐mouse (1:10,000, LI‐COR, Lincoln, NE, USA, #925‐32212) and Alexa Fluor anti‐rabbit 680 (1:10,000, ThermoFisher Scientific, #A27042). Fluorescence was quantified using the LiCor Odyssey imager.

### Data analysis

Unless otherwise noted, all data are reported as mean ± SEM. Student's *t*‐test was performed for comparison of two groups. ANOVA followed by the appropriate post-hoc analysis was performed for comparisons of 3 or more groups. A probability value of *P* ≤ 0.05 was considered significantly different. Statistical calculations were performed using the GraphPad Prism software (La Jolla, CA, USA). The CalR software was used to extract the averaged data for the metabolic parameters measured in the CLAMS at 30 °C and 4 °C during light and dark cycles respectively. The hourly plots for body temperature were also extracted from CalR^45^.

### Supplementary Information

Supplementary Information.

## Data Availability

The data that support the findings of this study are openly available in NCBI GEO at https://www.ncbi.nlm.nih.gov/geo/query/acc.cgi?acc=GSE227060, reference number GSE227060.
