# Supplementary material for: ATF4 expression in thermogenic adipocytes is required for cold-induced thermogenesis in mice via FGF21-independent mechanisms
Source: Sci Rep. 2024 Jan 18;14:1563. doi: 10.1038/s41598-024-52004-8 (PMC10796914; doi:10.1038/s41598-024-52004-8)
Supplement: Supplementary file 1 — Supplementary Information. [file 41598_2024_52004_MOESM1_ESM.pdf]

ATF4 Expression in Thermogenic Adipocytes is Required for Cold-Induced Thermogenesis in Mice via FGF21-Independent Mechanisms

Supplemental Experimental Procedures and Figure Legends

Sarah H. Bjorkman[1,2*]; Alex Marti[1*]; Jayashree Jena[1]; Luis Miguel García-Peña[1]; Eric T. Weatherford[1]; Kevin Kato[1], Jivan Koneru[1], Jason Chen[1], Ayushi Sood[1], Matthew J. Potthoff[1,3]; Christopher M. Adams[1,4]; E. Dale Abel[1,5]; Renata O. Pereira[1]

[1]Fraternal Order of Eagles Diabetes Research Center and Division of Endocrinology and Metabolism, Roy J. and Lucille A. Carver College of Medicine, University of Iowa, Iowa City, IA, USA.

[2]Department of Obstetrics and Gynecology, Reproductive Endocrinology and Infertility, University of Iowa Hospital and Clinics, Iowa City, IA, USA

[3]Department of Neuroscience and Pharmacology, Roy J. and Lucille A. Carver College of Medicine, University of Iowa, Iowa City, IA, USA.

[4]Division of Endocrinology, Diabetes, Metabolism and Nutrition, Department of Medicine, Mayo Clinic, Rochester, Minnesota, USA.

[5]Department of Medicine, David Geffen School of Medicine, University of California, Los Angeles, Los Angeles, CA, USA

Corresponding author: renata-pereira@uiowa.edu

Address: 169 Newton Road, 4338 PBDB | Iowa City, IA 52242

Phone: 319-335-9615

Supplemental Figure Legends:

Supplemental Figure 1: A-B. Data collected in BAT of WT mice exposed to 30 ˚C or 4 ˚C for 3 days. **A.** mRNA expression of thermogenic genes in iWAT normalized to *Tbp* (30 ˚C n=4; 4 ˚C n=4). **B.** mRNA expression of *Atf4*, *Fgf21* and *Gdf15* in iWAT normalized to *Tbp* (30 ˚C n=4; 4 ˚C n=4). **C.** mRNA expression of *Atf4* normalized to *Tbp* in the kidney, brain, mammary gland and in the eye of WT and ATF4 BKO female mice (WT n=6; KO n=5). Data are expressed as means ± SEM. Significant differences were determined by Student's *t*-test, using a significance level of $p<0.05$. * $p < 0.05$; ** $p < 0.01$; *** $p < 0.001$ significantly different vs. WT mice at 30 ˚C.

Supplemental Figure 2: Non-normalized tissue weights in WT and ATF4 BKO male mice. **A.** Brown adipose tissue (BAT) mass. **B.** Inguinal white adipose tissue (iWAT) mass. **C.** Gonadal white adipose tissue (gWAT) mass (WT n=6; KO n=5). Data are expressed as means ± SEM. Significant differences were determined by Student's *t*-test, using a significance level of $P < 0.05$.

Supplemental Figure 3: A. Principal component analysis (PCA) for the RNASeq data collected in BAT of cold exposed WT (n=2) and ATF4 BKO mice (n=3). **A.** mRNA expression of amino acid metabolism genes normalized to *Tbp* expression in BAT of FGF21 BKO mice cold exposed for 3 days. Data are expressed as means ± SEM. Significant differences were determined by Student's *t*-test, using a significance level of $P < 0.05$.

Supplemental Figure 4: mRNA expression of amino acid metabolism genes normalized to *Tbp* expression in BAT of ATF4 BKO mice cold exposed for 4 hours following fasting (WT=7; KO=4). Data are expressed as means ± SEM. Significant differences were determined by Student's *t*-test, using a significance level of $P < 0.05$.

Supplemental Figure 5: Full immunoblot image for Figure 1A. Bands used in the manuscript are highlighted by the red boxes. Immunoblot membranes were cut prior to hybridization with antibodies.

Supplemental Figure 6: Full immunoblot image for Figure 2E. Bands used in the manuscript are highlighted by the red boxes. Immunoblot membranes were cut prior to hybridization with antibodies.

Supplemental Figure 7: A. Full immunoblot image for Figure 3J. **A.** Full immunoblot image for Figure 3M. Bands used in the manuscript are highlighted by the red boxes. Immunoblot membranes were cut prior to hybridization with antibodies.

Supplemental Figure 8: Full immunoblot image for Figure 4E. Bands used in the manuscript are highlighted by the red boxes. Immunoblot membranes were cut prior to hybridization with antibodies.

Supplemental Figure 9: A. Full immunoblot image for Figure 5L. **A.** Full immunoblot image for Figure 5O. Bands used in the manuscript are highlighted by the red boxes. Immunoblot membranes were cut prior to hybridization with antibodies.

Supplemental Figure 10: Full immunoblot image for Figure 6D. Bands used in the manuscript are highlighted by the red boxes. Immunoblot membranes were cut prior to hybridization with antibodies.

Supplemental Figure 11: A. Full immunoblot image for Figure 7E. **A.** Full immunoblot image for Figure 7K. Bands used in the manuscript are highlighted by the red boxes. Immunoblot membranes were cut prior to hybridization with antibodies.

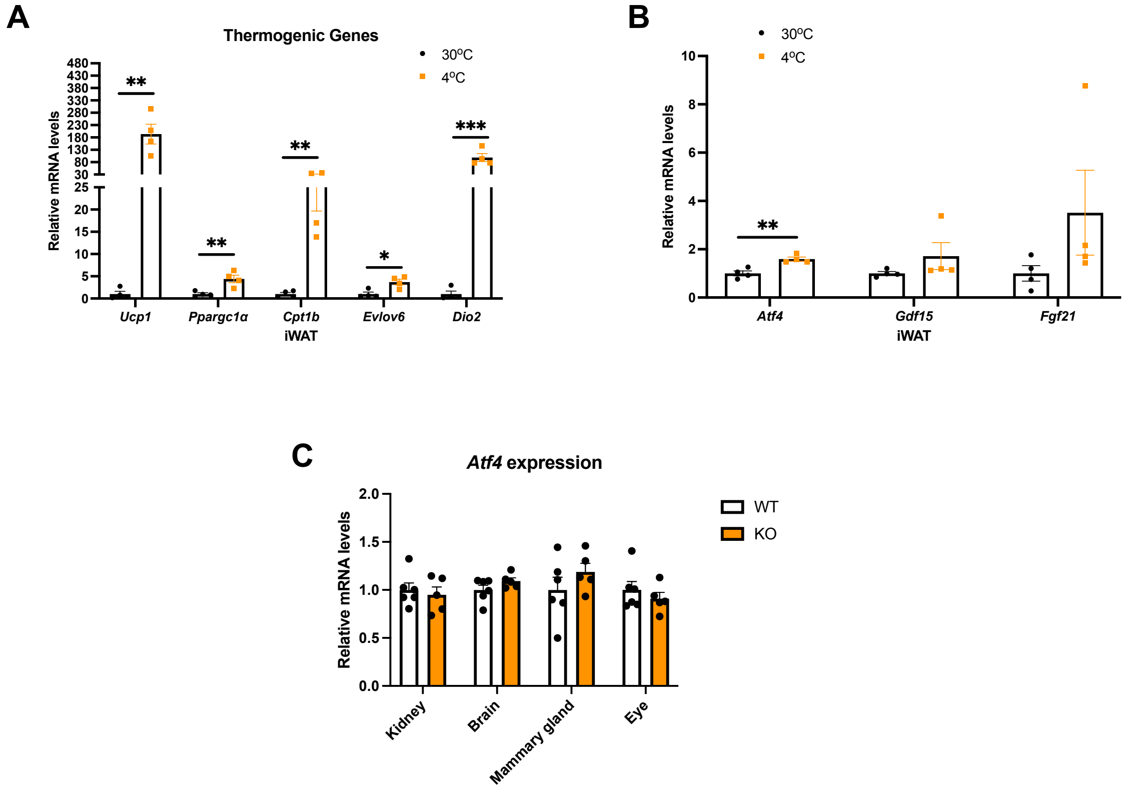

Supplemental Figure 1

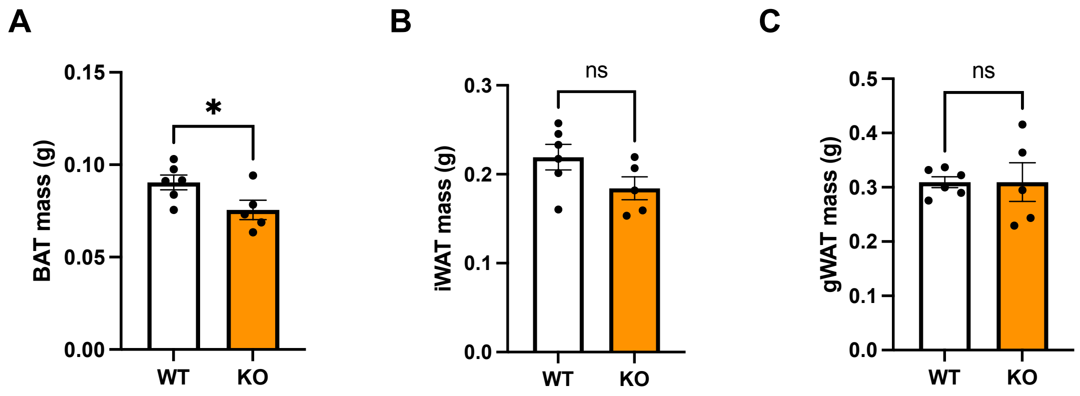

Supplemental Figure 2

A

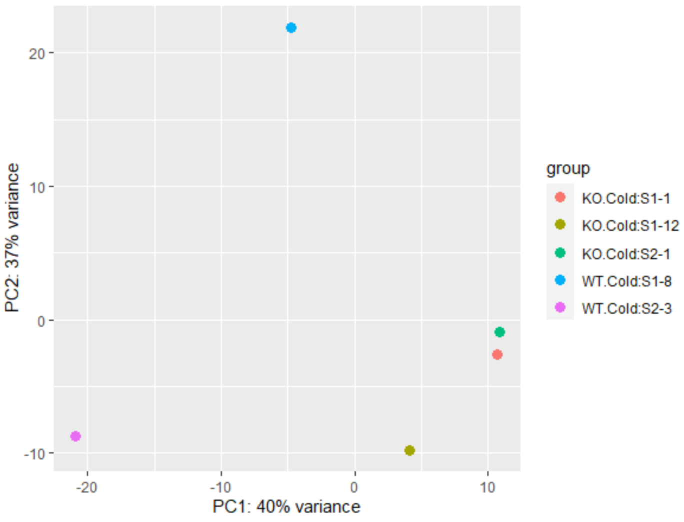

B

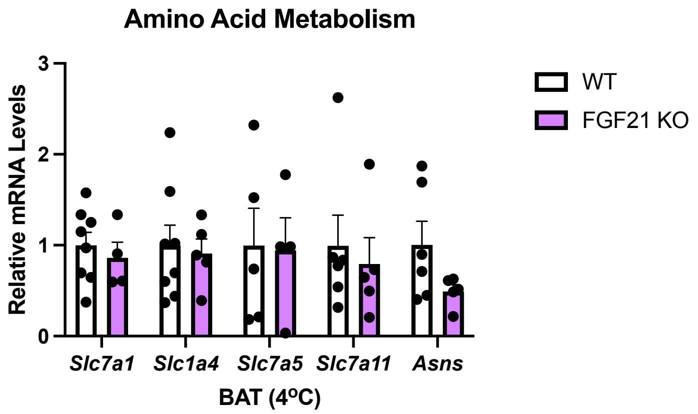

Supplemental Figure 3

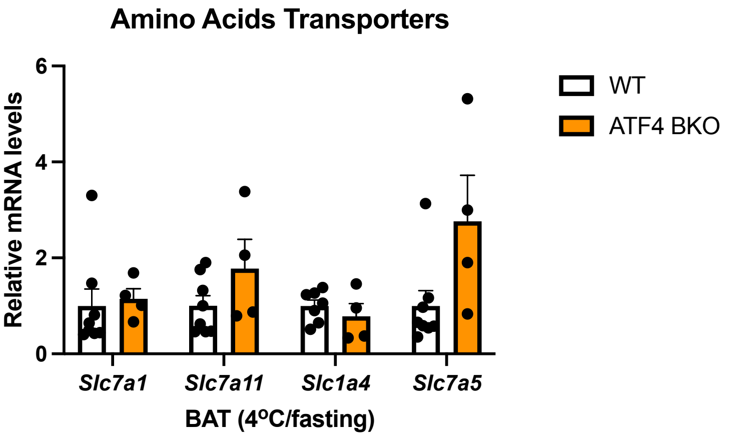

Supplemental Figure 4

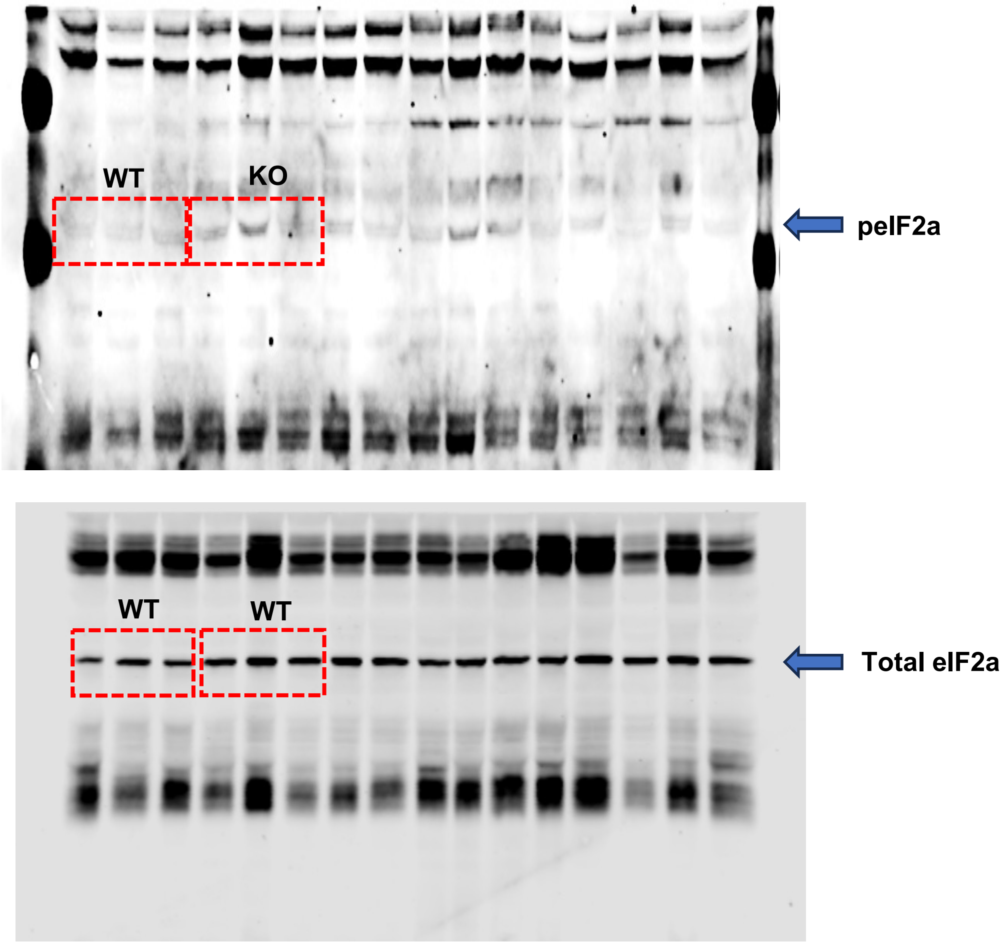

Supplemental Figure 5

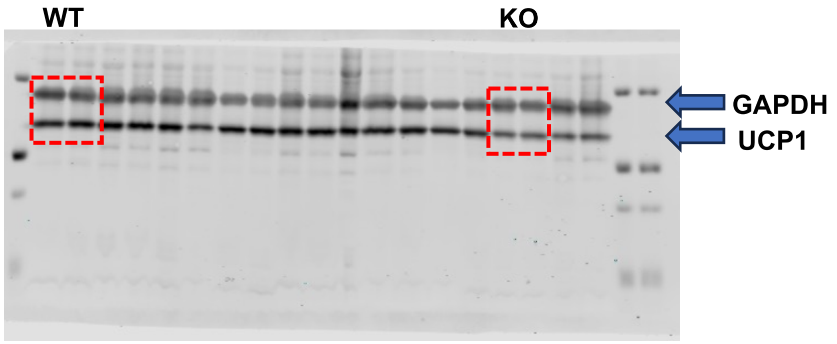

Supplemental Figure 6

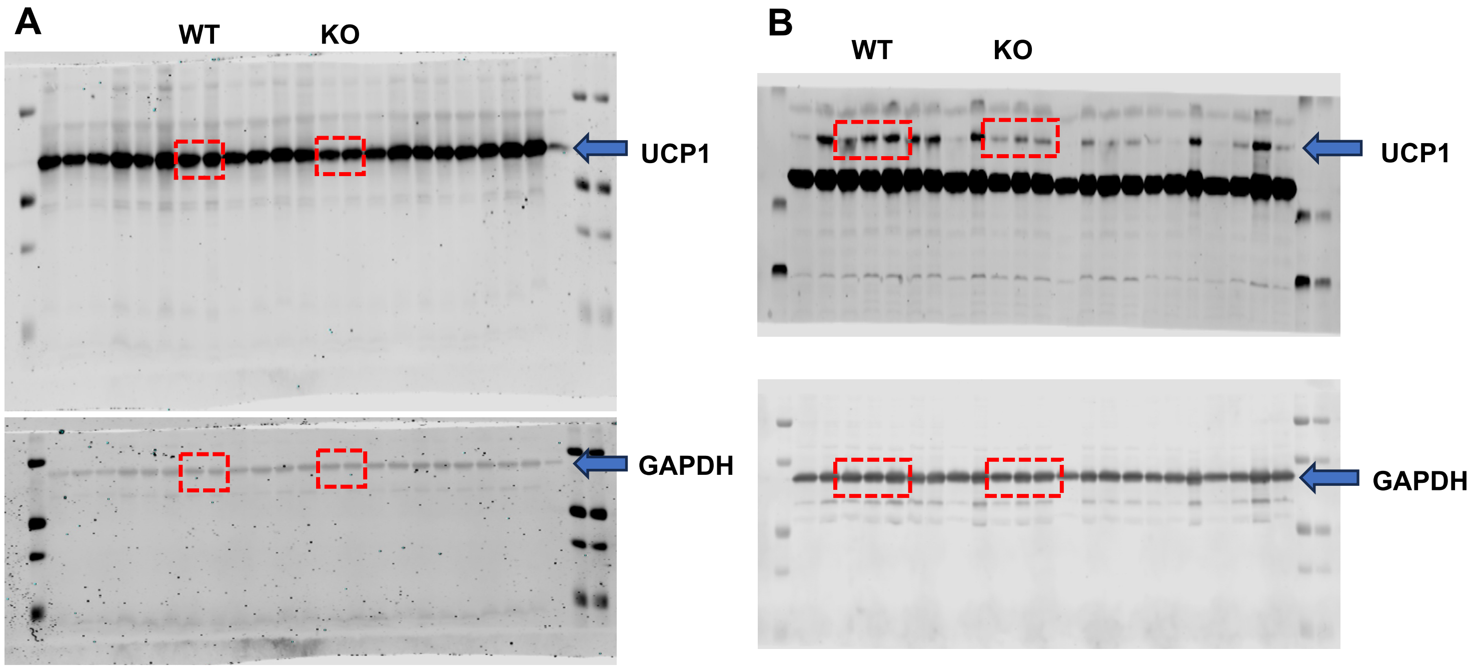

Supplemental Figure 7

A

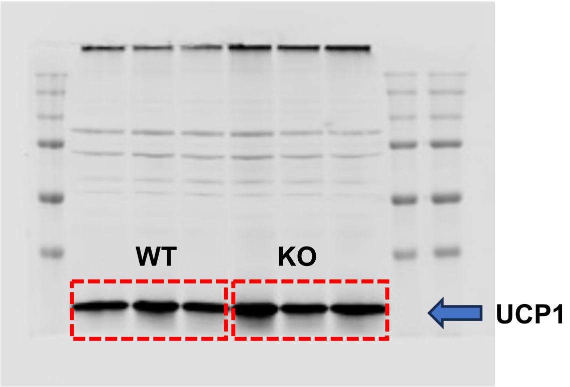

B

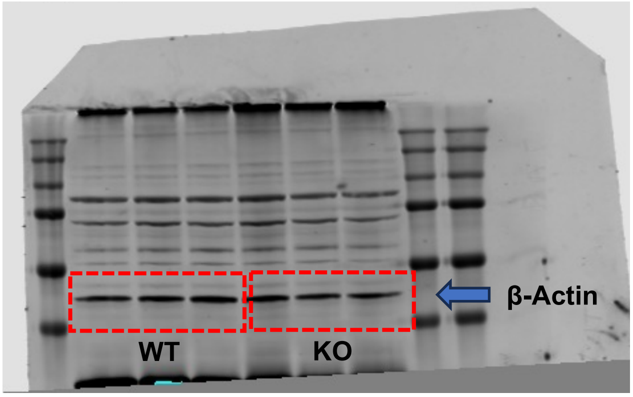

Supplemental Figure 8

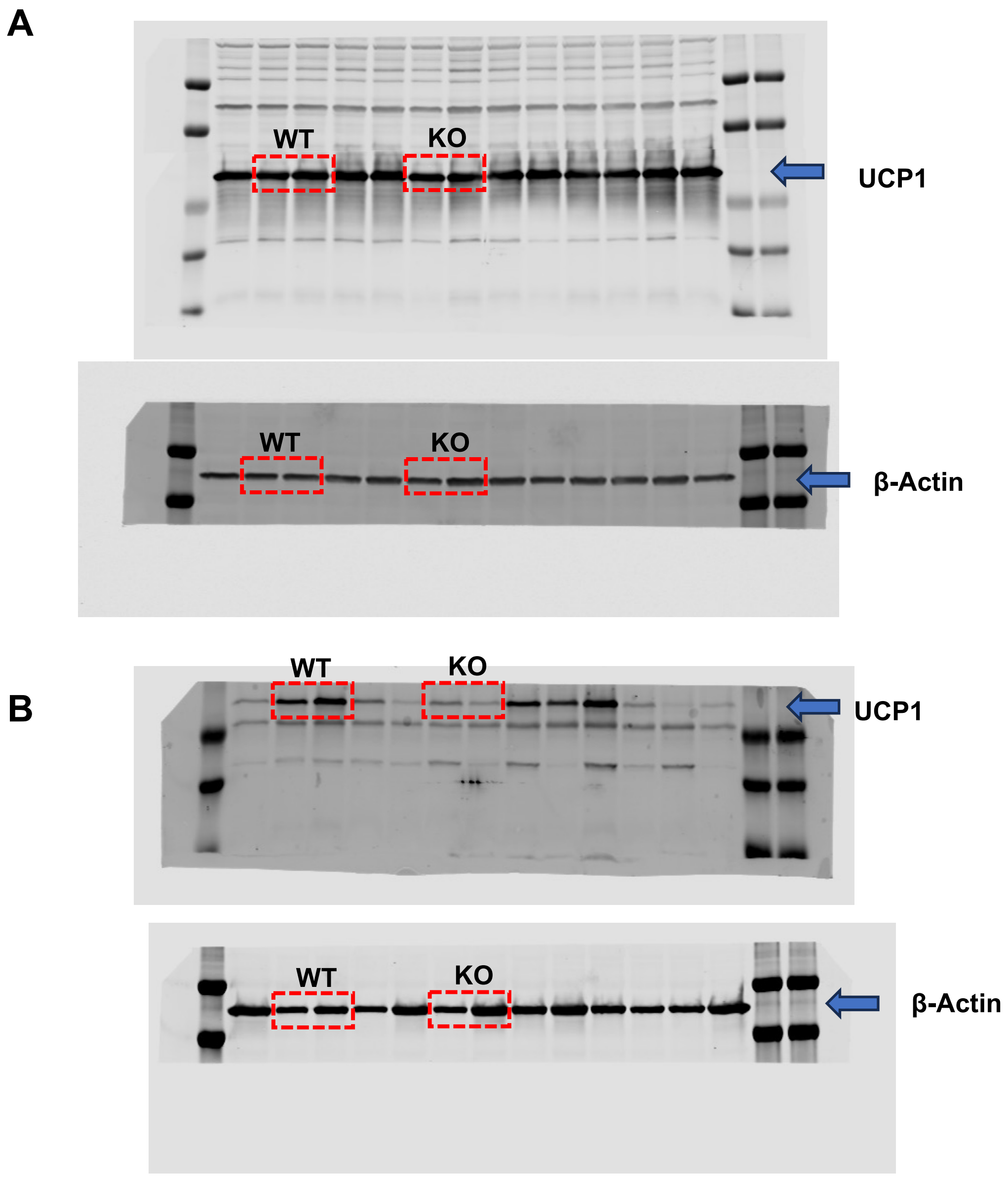

Supplemental Figure 9

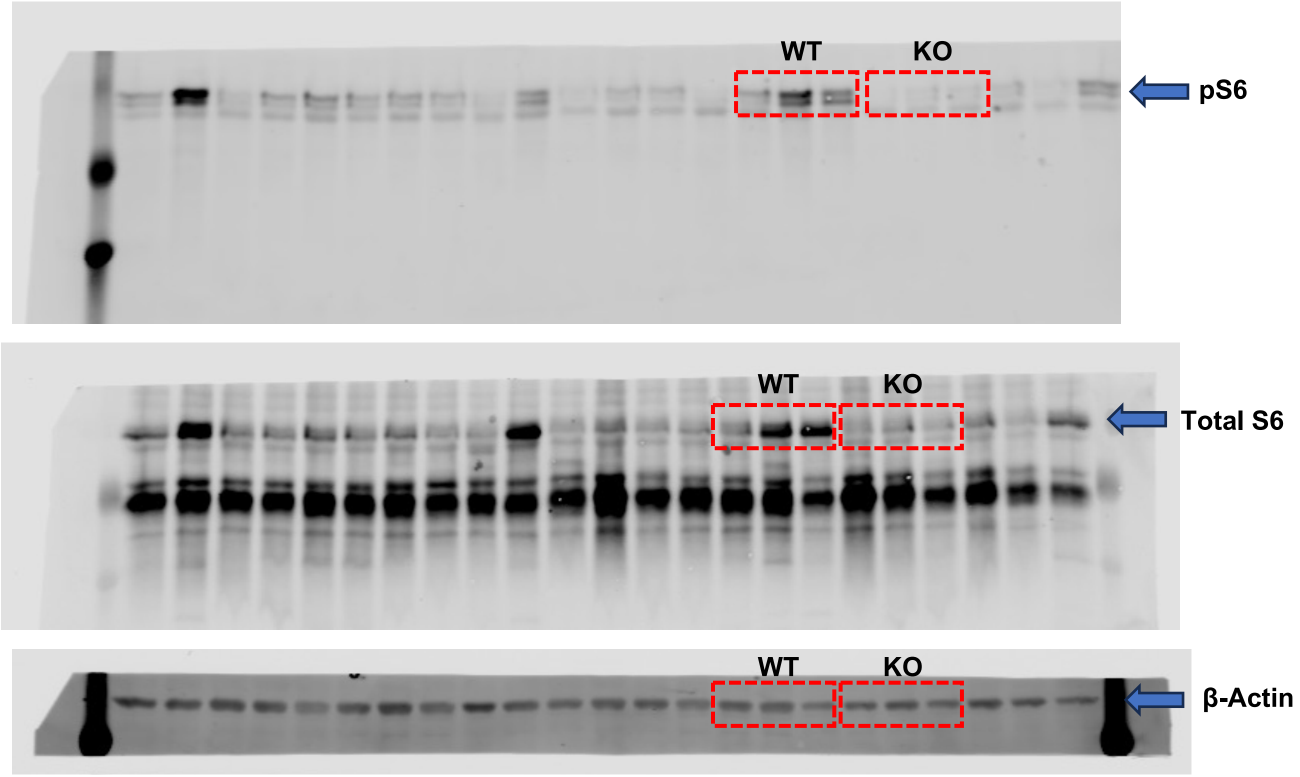

Supplemental Figure 10

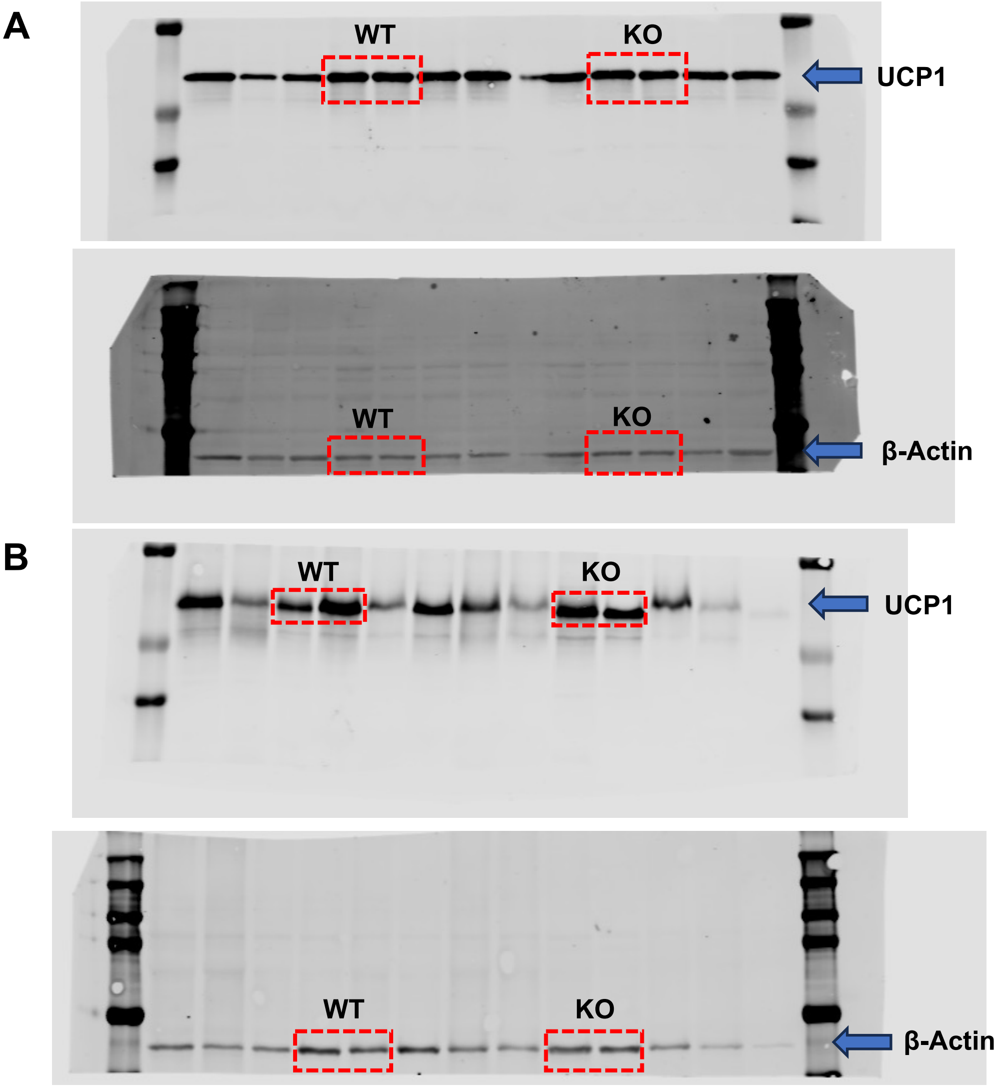

Supplemental Figure 11
